# An uncertainty-based interpretable deep learning framework for predicting breast cancer outcome

**DOI:** 10.1186/s12859-024-05716-7

**Published:** 2024-02-29

**Authors:** Hua Chai, Siyin Lin, Junqi Lin, Minfan He, Yuedong Yang, Yongzhong OuYang, Huiying Zhao

**Affiliations:** 1 School of Mathematics and Big Data, Foshan University, Foshan, 528000 China; 2 School of Data and Computer Science, Sun Yat-Sen University, Guangzhou, 510000 China; 3 Department of Medical Research Center, Sun Yat-Sen Memorial Hospital, Sun Yat-Sen University, Guangzhou, 510000 China

**Keywords:** Prognosis analysis, Deep learning, Breast cancer, Survival analysis

## Abstract

**Background:**

Predicting outcome of breast cancer is important for selecting appropriate treatments and prolonging the survival periods of patients. Recently, different deep learning-based methods have been carefully designed for cancer outcome prediction. However, the application of these methods is still challenged by interpretability. In this study, we proposed a novel multitask deep neural network called UISNet to predict the outcome of breast cancer. The UISNet is able to interpret the importance of features for the prediction model via an uncertainty-based integrated gradients algorithm. UISNet improved the prediction by introducing prior biological pathway knowledge and utilizing patient heterogeneity information.

**Results:**

The model was tested in seven public datasets of breast cancer, and showed better performance (average C-index = 0.691) than the state-of-the-art methods (average C-index = 0.650, ranged from 0.619 to 0.677). Importantly, the UISNet identified 20 genes as associated with breast cancer, among which 11 have been proven to be associated with breast cancer by previous studies, and others are novel findings of this study.

**Conclusions:**

Our proposed method is accurate and robust in predicting breast cancer outcomes, and it is an effective way to identify breast cancer-associated genes. The method codes are available at: https://github.com/chh171/UISNet.

**Supplementary Information:**

The online version contains supplementary material available at 10.1186/s12859-024-05716-7.

## Background

According to the global cancer statistics in 2020, breast cancer is the most common malignant tumor, accounting for two million (11.7%) patients worldwide [1]. The outcomes of these breast cancer patients were observed to be significantly different under the same treatment, reflecting the heterogeneity of breast cancer. Accurate breast cancer outcome prediction is important for designing effective follow-up treatments, and improving the survival periods and life quality of patients [2].

With the advancement of molecular sequencing techniques, an increasing amount of high-dimensional genomic data has been used to evaluate cancer outcomes to support clinical decision- making [3, 4]. The most widely used method for evaluating patient risks is the Cox proportional hazard model [5]. This method analyses the influences of different factors on cancers by calculating the survival ratios of patients without knowing patients’ survival distributions. In addition, Wang et al. designed random survival forests (RSF) to predict cancer outcomes by utilizing the bootstrapping strategy [6]. However, these methods have limited performance on high-dimensional gene expression data [7]. To solve this problem, many feature dimensionality reduction technologies were added to the algorithm. Lin used features extracted by principal component analysis (PCA) in the Cox method to predict disease prognosis [8]. Considering that PCA performs poorly in a high-dimensional nonlinear space, Cai selected kernel-PCA instead of PCA to generate compressed features for patient risk prediction [9]. Another way to solve the computational challenge caused by high-dimensional features is to add a regularization component to the Cox model. Boulesteix combined adaptive Lasso regularization and the Cox regression method (IPW-lasso) to estimate cancer prognosis [10]. The method minimized the likelihood function via an L1-norm regularization constant to shrink the coefficients of the features. In addition, the survival support vector machine (SSVM) proposed by Evers yielded improved cancer outcome prediction performance by using a sparse kernel function. However, choosing a suitable kernel function and hyperparameters is a complex process. Recently, Liu et al. designed an integrated learning method called EXSA based on the XGBoost framework to predict cancer outcomes. The results show that it outperformed other traditional machine learning methods [11].

In recent years, deep learning (DL) technologies have demonstrated their efficacy in handling high-dimensional nonlinear features [12]. The residual neural network was used in Li’s work for breast cancer prognostic index classification [13]. Deep_surv, proposed by Katzman, was designed to estimate cancer outcomes by combining a deep neural network (DNN) and the proportional hazard loss function [14]. Chaudhary used an Autoencoder to reconstruct high-dimensional expression features, and the generated features were used for liver cancer survival analysis [15]. Based on this method, Yang et al. proposed DCAP by using a denoising autoencoder to build a robust model for defending against data noise [16]. Nonetheless, the separation of the feature extraction and risk prediction processes hindered the convenience of this method. To solve this problem, an end-to-end framework called TCAP was designed to combine the risk prediction loss and data recovery loss [17]. Bashier et al. proposed a multi-omics data integration approach that combines gene similarity networks with convolutional neural networks to accurately predict the stage of breast tumors [18]. On the basis of these studies, to speed up the convergence of DNN model and reduce the risk of overfitting during model training, Qiu introduced a meta-learning-based network for cancer outcome prediction [19].

Although DL-based methods have achieved better results in cancer outcome prediction, the application of these methods is still limited by the lack of model interpretability. Interpreting the factors associated with cancer outcomes is important for medical decision-making and target drug development. The widely used method for solving this problem is differential expression analysis (DEA). Nevertheless, when the average expression of the given features is low, the log-fold change values computed in DEA are disproportionately affected by noise [20]. Hence, Hao proposed an interpretable DNN framework for cancer survival analysis, by calculating the gradients in the neural network [21]. However, this approach may lead to gradient saturation, making it difficult for the neural network to identify important features [22]. Zhao et al. proposed DeepOmix for cancer prognosis prediction [23]. According to the predicted risks, DeepOmix performed the Kolmogorov‒Smirnov test to identify prognosis-related genes. However, the genes identified by these methods are hard to be proved as effectively related to cancer outcomes. The studies indicated that by removing these genes from the model, the accuracy of prediction is not reduced dramatically [24]. Thus, it is necessary to develop an interpretable model to accurately predict cancer outcomes and identify cancer-related key genes to reveal the novel cancer mechanisms.

To address these problems, we propose an Uncertainty-based Interpretable deep Semi-supervised Network (UISNet) for breast cancer outcome prediction. The main contributions of our research are given as follows:

1. An uncertainty-based algorithm that combines the Monte Carlo dropout and the integrated gradients is designed to improve the reliability of the interpretable results.
2. By introducing the prior biological pathway information as a sparse layer, UISNet deals with the high-dimensional gene expression data effectively.
3. UISNet considered the heterogeneity of patients to extract useful information for cancer outcome prediction. This information was integrated into a unified framework after dimension reduction. All these tasks are simultaneously optimized by the shared representations in the neural network.
4. UISNet was used in seven breast cancer datasets from the TCAG and GEO databases, and the prediction results were analyzed comprehensively. The results indicated that UISNet is accurate and robust when predicting breast cancer outcomes, and is able to identify prognosis-related genes, efficiently.

The details of UISNet are introduced in Section "Methods". Section "Results" shows the experimental results and biological analysis. Finally, we present the conclusion and discussion in Section "Discussion".

## Methods

### Datasets

In this study, seven breast cancer datasets collected from GEO (https://www.ncbi.nlm.nih.gov) and TCGA (https://www.cancer.gov/about-nci/organization/ccg/research/structural-genomics/tcga) were used for method evaluations. Considering the requirement for cancer outcome prediction data with consistent gene expression profiles and comprehensive information on patient survival, we identified six available datasets from the GEO database, encompassing a total of 1323 breast cancer patients. The features of 4767 genes in these datasets were normalized by log transformation, and the batch effect was removed by using the “*limma*” package [25]. The statistical information of each used dataset is given in Table 1.

**Table 1:** The statistical information of the utilized cancer data in our study

Dataset	Censored patients	Sample size	Censored rate (%)
BRCA	530	609	87.03
GSE2990 [26]	77	125	61.60
GSE9195 [27]	65	77	84.42
GSE11121 [28]	154	200	77.00
GSE17705 [29]	227	298	76.17
GSE19615 [30]	14	115	12.17
GSE25066 [31]	397	508	78.15
BRCA_all	1464	1932	75.78

### The architecture of the proposed deep learning framework

**Fig. 1:**
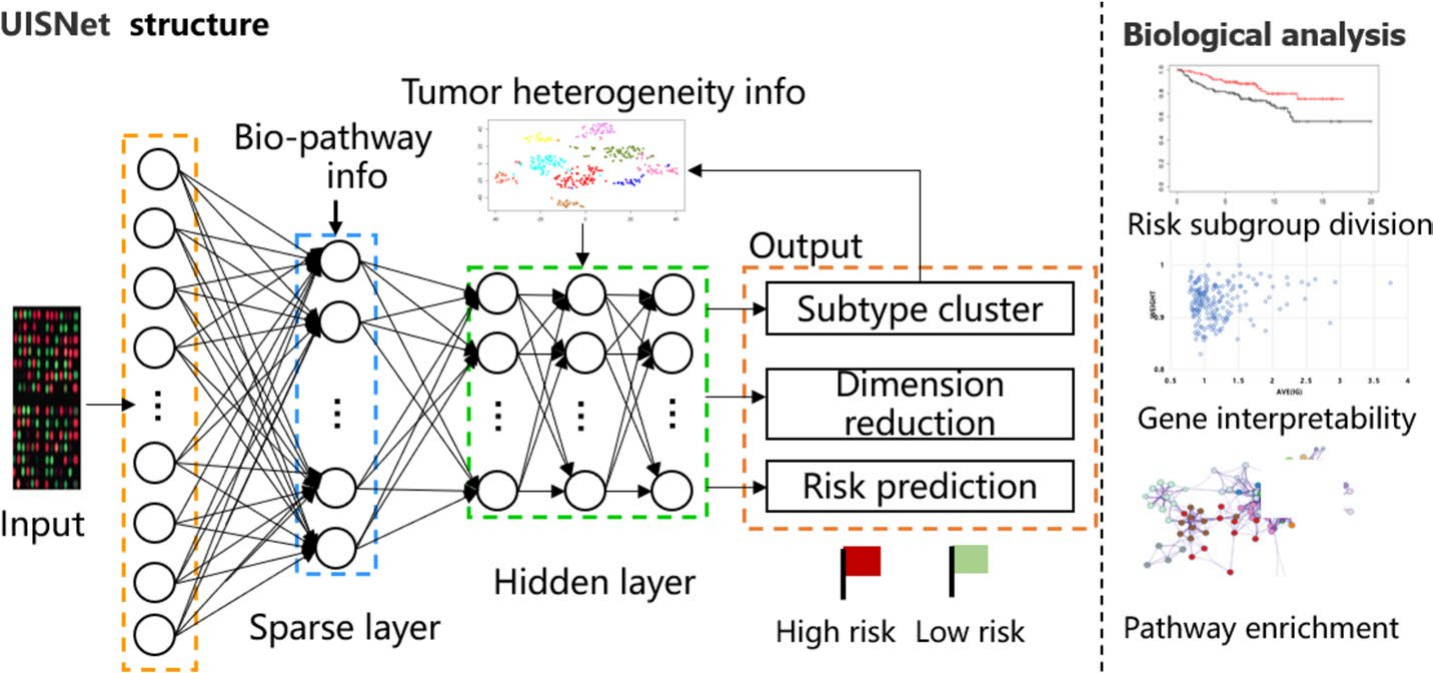
The architecture of UISNet for breast cancer outcome evaluation. Prior biological pathway information is introduced in the sparse layer, and UISNet predicts breast cancer outcomes by integrating patient heterogeneity clustering, dimensionality reduction, and cancer outcome prediction tasks into a unified framework

As shown in Fig. 1, high-dimensional gene expression data are given in the input layer, and prior biological pathway information is introduced in the sparse layer. The uncertainty-based interpretable deep semi-supervised network (UISNet) can learn meaningful information by incorporating the prior biological knowledge in the sparse layer (e.g., KEGG and Reactome gene connection pathways). The knowledge regarding the learned relationships between genes and functional pathways is used to form sparse connections between the input layer and the sparse layer instead of full connections.

Here, we constructed a binary biadjacency matrix to represent the sparse connections between the genes and functional pathways by using the strategy presented by Hao [21]. Supposing that *p* gene features and prior information, including *q* pathways from the KEGG and Reactome databases, are given in the neural network, the binary biadjacency matrix can be expressed as $A\epsilon {\mathbb{B}}^{q\times p}$, and the element ${a}_{ij}$ in $A$ is given as:

1 $$a_{ij} = \left\{ {\begin{array}{*{20}l} {1,} \hfill & {1 \le i \le q,1 \le j \le p} \hfill \\ {0,} \hfill & {else} \hfill \\ \end{array} } \right.$$

The node values *h* in the UISNet framework are computed as follows:

2 $$h_{l + 1} = \left\{ {\begin{array}{*{20}l} {Relu\left( {\left( {W*A} \right)h_{gene} + b} \right),} \hfill & {sparse\;layer } \hfill \\ {Relu\left( {W*h_{l} + b} \right),} \hfill & {hidden\;layer} \hfill \\ \end{array} } \right.$$

where *RelU* is a nonlinear activation function, ${h}_{gene}$ represents the gene expression values, ${h}_{l}$ is the output in layer *l*, *W* is the weight matrix, and *b* is the bias.

The feature dimensionality reduction is performed by the Eq. (3), where $X=\left({x}_{1},{x}_{2},\dots {x}_{p}\right)$ represents the gene expressions of the breast cancer patients, and *Z* is the low-dimensional representation of *X* in the last hidden layer. The feature dimensionality reduction task is used to obtain high-quality compressed features in the hidden layer. Similar to the encoder-decoder structure, supposing that *E* is the encoder function and *D* is the decoder function, the compressed *Z* is written as: *Z* = *E*(*X*), and the recovered *X′* can be expressed as *X′* = *D*(*Z*). The loss induced by the dimensionality reduction task is written as:

3 $${L}_{D}={\sum }_{i=1}^{p}{\left({x}_{i}-{x}_{i}^{\mathrm{^{\prime}}}\right)}^{2}$$

The subtype clustering task is designed to extract information on breast cancer patient heterogeneity. In the last hidden layer, a feature matrix is formed by integrating the produced *Z* and the cluster labels *L*. The clustering task loss in UISNet is defined as the KL divergence between the two distributions *S* and *T* [32]:

4 $${L}_{c}=KL(S||T)=\sum_{i}\sum_{j}{s}_{ij}log\frac{{s}_{ij}}{{t}_{ij}}$$

where ${t}_{ij}$ describes the similarity between the cluster center ${\mu }_{j}$ and an embedded point ${z}_{j}$ by Student’s t-distribution:

5 $${t}_{ij}=\frac{{\left(1+{\Vert {z}_{i}-{\mu }_{j}\Vert }^{2}\right)}^{-1}}{{\sum }_{j}{\left(1+{\Vert {z}_{i}-{\mu }_{j}\Vert }^{2}\right)}^{-1}}$$

${s}_{ij}$ is the target distribution:

6 $${s}_{ij}=\frac{{t}_{ij}^{2}/{\sum }_{i}{t}_{ij}}{{\sum }_{j}\left({t}_{ij}^{2}/{\sum }_{i}{t}_{ij}\right)}$$

The initial cluster labels *L* of the patients are calculated by k-means. The number of clusters *k* is the value in [2–4] with the largest silhouette score. To ensure the accuracy of the clustering task in UISNet, the computed labels are updated in each epoch while the program runs.

The risk prediction task is used to evaluate breast cancer prognoses by Eq. (7). In Eq. (7), $S\left(t\right)=Pr(T\ge t)$ is the survival probability that a patient will survive before time $t$. The time interval $T$ is the time elapsed between data collection and the patient’s last contact (the end of the experiment/patient death). The risk function at *t* can be given as follows:

7 $$\lambda \left(t\right)=\underset{\delta \to 0}{{\text{lim}}}\frac{{\text{Pr}}(t\le T<t+\delta |T\ge t)}{\delta }$$

The loss function of the outcome prediction task can be expressed as Eq. (8) [14]:

8 $${L}_{P}=-\sum_{i=1}\left({h}_{\theta }\left(x\right)-log\sum_{j\in \mathfrak{R}\left({T}_{i}\right)}{exp}^{{h}_{\theta }\left({x}_{j}\right)}\right)$$

where UISNet updates $h\left(x\right)$ according to the weight $\theta$, and $\mathfrak{R}\left({T}_{i}\right)$ represents the risk set of the breast cancer patients that are still alive at time point ${T}_{i}$.

By integrating the feature dimensionality reduction, patient heterogeneity clustering, and cancer outcome prediction tasks into a unified framework, the total loss of UISNet can be given as follows:

9 $${l}_{UISNet }={\gamma L}_{D}+{\beta L}_{C}+{L}_{P}$$

$\gamma$ and $\beta$ can balance the importance of these tasks, which can be seen as the hyperparameters chosen by the cross-validation step. In this study, the value of $\gamma$ was set to 1, and $\beta$ was set to 10.

### The uncertainty-based integrated gradients algorithm

In [21], the gradients of the output *y* with respect to the input *x* $(W=\partial y/\partial x)$ were used to quantify the importance of each gene to cancer prognosis. However, computing the gradients in a deep neural network may lead to gradient saturation. To interpret the feature importance of the prediction model, UISNet uses the Gauss‒Legendre quadrature to approximate the integral of the gradients after calculating the gradients of the input *x* across different scales against the baseline ${x}_{i}$ (zero-scaled):

10 $$IG\left({x}_{i}\right)\colon\colon =\left({x}_{i}-{x}_{i}^{\prime}\right)\times {\int }_{\alpha =0}^{1}\frac{\partial F\left({x}^{\prime}+\alpha \times (x-{x}^{\prime})\right)}{\partial {x}_{i}}d\alpha$$

Nevertheless, the evaluation results given by the integrated gradients algorithm are not always reliable. Calculating the uncertainty of the predictions can enable the reliability of the results to be judged. Bayesian neural networks have been designed to quantify the uncertainty of results, but due to the large number of required computations, Monte Carlo dropout and Gaussian distribution models are often used as approximate solutions for Bayesian neural networks. Compared to Gaussian distribution models, Monte Carlo dropout can better approximate Bayesian neural networks by using the dropout term as one regularization term, for calculating the uncertainty of the results [33]. The objective function for using L2 regularization in Monte Carlo dropout can be expressed as:

11 $${l}_{MC}:=\frac{1}{n}{\sum }_{1}^{n}E\left({y}_{i},{\widehat{y}}_{i}\right)+\lambda {\sum }_{l=1}^{L}{\Vert {W}_{i}\Vert }_{2}^{2}+{\Vert {b}_{i}\Vert }_{2}^{2}$$

where *L* is the number of layers in the deep neural network, and ${y}_{i}$ and ${\widehat{y}}_{i}$ are the target and the output of the network, respectively. By following [34], we trained UISNet with different dropout settings at *T* inference times as the Monte Carlo dropout strategy. Supposing that *lgx*_*i*_ represents the integrated gradients importance of the *i*th node, the uncertainty of the *i*th features is designed as:

12 $$U\left({x}_{i}\right)=std\sum lg{x}_{i}^{t}/ ave\sum lg{x}_{i}^{t}$$

where *std*(*) and *ave*(*) are the standard deviation and average values of $lg{x}_{i}$ at *T* inference times, respectively. The importance weight of the gene feature in the network is expressed as:

13 $$V\left({x}_{i}\right)=\left(1-{U}{\prime}\left({x}_{i}\right)\right)* IG\left({x}_{i}\right)$$

where *U′* $\left({x}_{i}\right)$ is the adjusted uncertainty value of $U\left({x}_{i}\right)$ after log transformation and min–max normalization. In summary, the UISNet algorithm is given as follows:

**Figure:**
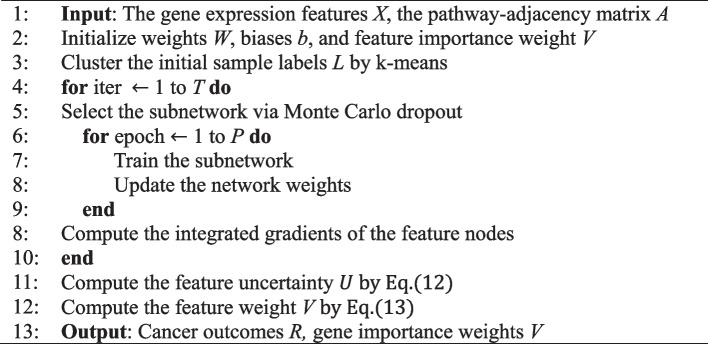
Algorithm of *UISNet*

### Performance evaluations and parameter selection

In this study, the cancer outcome prediction performances of different methods were compared through the C-index and |log10(P)| values. The C-index value is the fraction of all pairs of patients whose predicted outcomes are correctly ordered based on Harrell’s C statistics [35]. A higher |log10(P)| value indicates more significant survival differences between the patient subgroups divided based on the predicted risks (Additional file 1).

The parameter list in this study is given below. The number of nodes in hidden layer 1 was set to 1000, and the number of nodes in hidden layer 2 was set to 500. The number of nodes *Z* in hidden layer 3 was selected from [10, 20, 50]. The learning rate (LR) was selected from [1e-6,1e-7,1e-8], and the maximum number of epochs in the neural network was set to 2000. The optimal parameters were selected by fivefold cross validation. To access the robustness of our method in 5-fold cross-validation and 10-fold cross-validation, in Additional file 1; Supplementary Table S1 we show the deviation obtained by UISNet in 5-fold cross validation and 10-fold cross validation.

## Results

### Method comparison

The UISNet model was evaluated by the C-index (CI) in predicting the outcome of the patients in seven breast cancer datasets. The average CI values are given in Table 2. The UISNet was compared with six methods, the adaptive Lasso-penalized Cox model (IPW-lasso), the integrated learning-based Cox method (EXSA), the deep survival network (Deep_surv), the denoising autoencoder-based Cox model (DCAP) and the deep survival network with a meta-learning framework (MTC). As shown in Table 2, the UISNet achieved the average CI (0.691) across seven datasets, which is significantly higher than the CI 0.619, 0.638, 0.653, 0.665, and 0.677 achieved by the methods, IPF-lasso, EXSA, Deep_surv, DCAP, and MTC, respectively. The t-test was performed on the results obtained from UISNet and other methods, demonstrating significant improvements of our method compared to the alternative approaches.

**Table 2:** The CI values obtained by different methods on breast cancer datasets

Dataset	IPF-lasso	EXSA	Deep-surv	DCAP	MTC	UISNet
BRCA	0.629 (± 0.038)	0.637 (± 0.077)	0.664 (± 0.047)	0.671 (± 0.065)	0.678 (± 0.037)	0.694 (± 0.043)
GSE2990	0.545 (± 0.089)	0.570 (± 0.089)	0.563 (± 0.074)	0.577 (± 0.059)	0.596 (± 0.091)	0.596 (± 0.043)
GSE9195	0.657 (± 0.239)	0.695 (± 0.203)	0.701 (± 0.214)	0.712 (± 0.211)	0.755 (± 0.052)	0.753 (± 0.112)
GSE11121	0.641 (± 0.102)	0.669 (± 0.092)	0.680 (± 0.063)	0.681 (± 0.116)	0.695 (± 0.094)	0.727 (± 0.074)
GSE17705	0.633 (± 0.099)	0.626 (± 0.070)	0.659 (± 0.061)	0.670 (± 0.067)	0.682 (± 0.081)	0.687 (± 0.073)
GSE19615	0.640 (± 0.059)	0.649 (± 0.085)	0.674 (± 0.078)	0.688 (± 0.067)	0.711 (± 0.041)	0.703 (± 0.048)
GSE25066	0.623 (± 0.130)	0.651 (± 0.051)	0.663 (± 0.108)	0.681 (± 0.064)	0.672 (± 0.056)	0.706 (± 0.044)
BRCA_all	0.588 (± 0.107)	0.610 (± 0.049)	0.622 (± 0.044)	0.640 (± 0.065)	0.642 (± 0.041)	0.660 (± 0.031)
Average	0.619	0.638	0.653	0.665	0.677	0.691
P-value ^a^	5.49E-7	3.69E-7	9.5E-7	9.2E-5	0.039	-

^a^ The t-tests by comparisons with UISNet

**Fig. 2:**
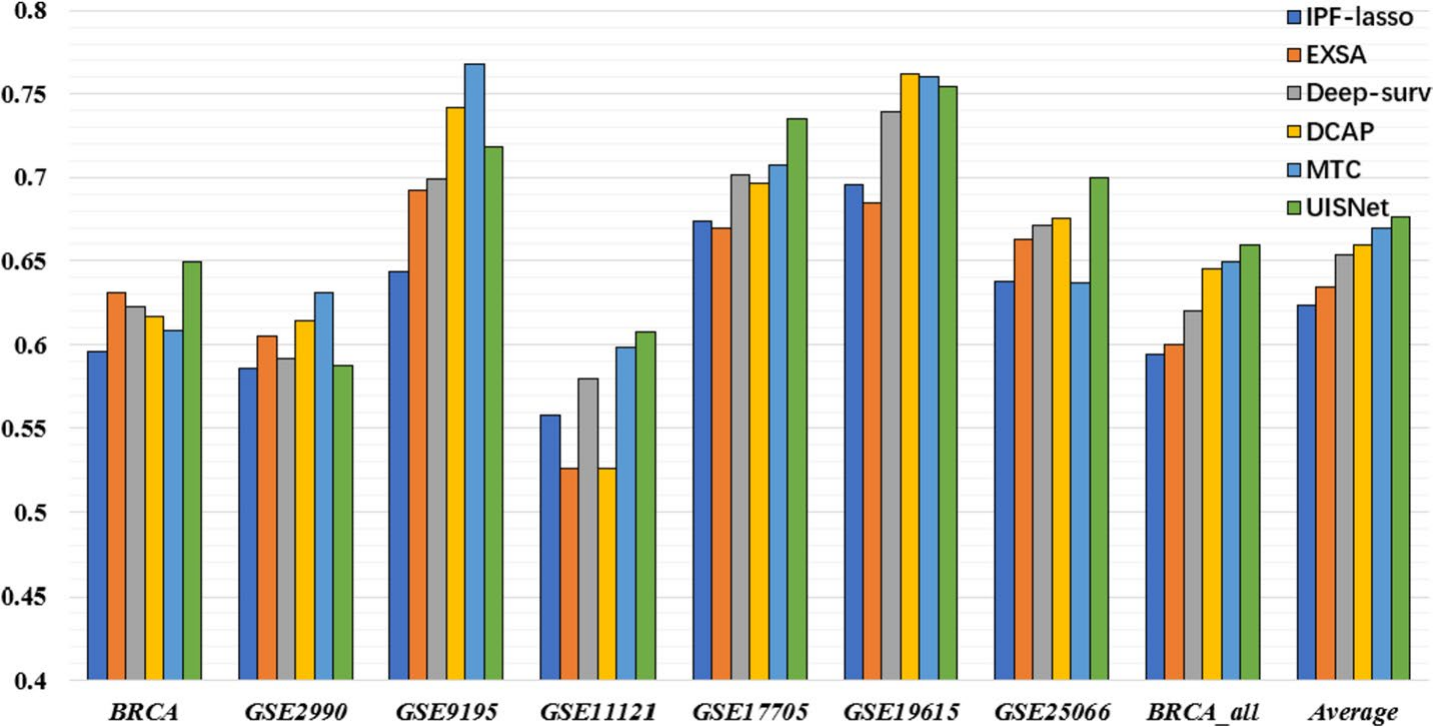
The AUC scores obtained by different methods on eight breast cancer datasets

When we divided the patients into subgroups according to the estimated prognosis risks, UISNet achieved average |log10(P)|= 1.608 across the seven datasets, which is higher than the |log10(P)| values achieved by IPF-lasso (1.036). Meanwhile, UISNet performed better than the other four compared methods (Table 3, average |log10(P)|= 1.167). Additionally, we show the average time-dependent AUC scores [36] in Fig. 2. By testing on the eight breast cancer datasets, the UISNet achieved the highest AUC score of 0.676 among the compared methods (IPF-lasso = 0.623, EXSA = 0.634, Deep-surv = 0.653, DCAP = 0.660, MTC = 0.670).

**Table 3:** The |log10(P)| values produced by different methods on breast cancer datasets

Dataset	IPF-lasso	EXSA	Deep-surv	DCAP	MTC	UISNet
BRCA	0.237 (± 0.254)	0.622 (± 0.502)	0.870 (± 0.425)	0.790 (± 0.757)	1.174 (± 0.412)	1.156 (± 0.461)
GSE2990	0.274 (± 0.272)	0.455 (± 0.794)	0.348 (± 0.239)	0.455 (± 0.206)	0.303 (± 0.267)	0.410 (± 0.314)
GSE9195	0.701 (± 0.467)	0.535 (± 0.560)	0.535 (± 0.560)	0.562 (± 0.551)	1.063 (± 0.120)	0.719 (± 0.494)
GSE11121	0.855 (± 0.896)	0.585 (± 0.693)	0.621 (± 0.510)	0.871 (± 0.912)	0.976 (± 0.849)	1.288 (± 0.761)
GSE17705	0.845 (± 0.678)	0.324 (± 0.333)	0.730 (± 0.323)	0.771 (± 0.262)	1.018 (± 0.756)	1.265 (± 0.868)
GSE19615	1.167 (± 1.208)	0.771 (± 0.509)	1.986 (± 1.605)	1.156 (± 0.637)	2.540 (± 1.043)	2.035 (± 1.462)
GSE25066	2.046 (± 2.032)	1.421 (± 0.356)	2.362 (± 2.082)	2.622 (± 1.825)	1.277 (± 0.823)	2.813 (± 1.514)
BRCA_all	2.163 (± 1.050)	2.071 (± 2.000)	1.944 (± 1.227)	2.728 (± 1.562)	2.851 (± 1.435)	3.174 (± 0.821)
Average	1.036	0.848	1.175	1.244	1.400	1.608

### Parameter sensitivity analysis

**Fig. 3:**
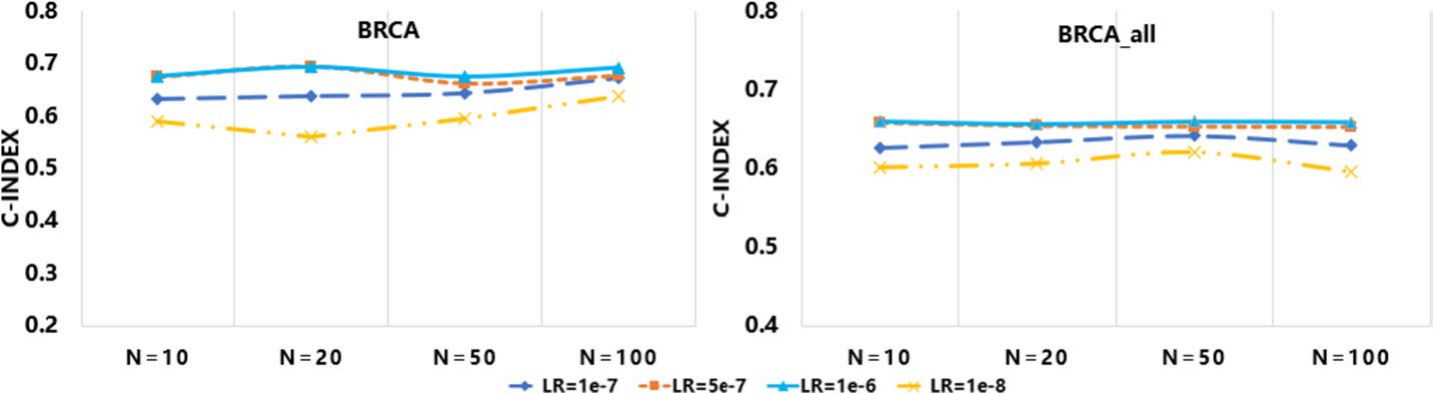
Parameter sensitivity analysis for UISNet. The y-axis values represent the C-index values obtained by using different hyperparameter combinations. The number of nodes in hidden layer 3 was selected from [100, 50, 20, 10], and the learning rate was set to [1e-6, 1e-7, 5e-7, 1e-8]

To evaluate the effects of the hyperparameters on the prediction of UISnet, we examined the CI values obtained on BRCA and BRCA_all with different parameter combinations (Fig. 3). The number of nodes in hidden layer 3 was selected from [100, 50, 20, 10], while the learning rate was set to [1e-6, 1e-7, 5e-7, 1e-8]. By comparing the standard deviation values of the CI values while one parameter was fixed, we found that the effect of the node size in the network was relatively small (*std* = 0.010), which was lower than the result of the learning rate (*std* = 0.028). Nevertheless, it is difficult to determine the optimal parameter combinations in different datasets. In this study, we used a fivefold CV to select suitable hyperparameters of UISNet in model training.

### Ablation experiment

**Fig. 4:**
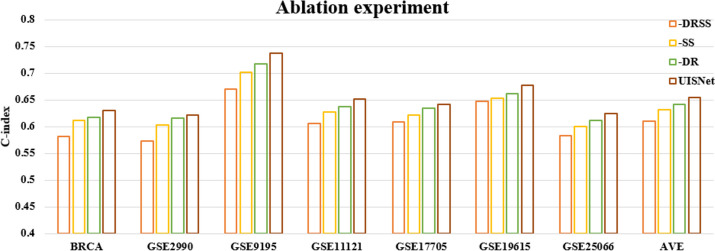
Ablation experiment results obtained by UISNet for cancer outcome prediction. -DRSS represents the result achieved by only using the cancer outcome prediction task, and -SS and -DR indicate the results obtained when excluding the clustering task and the dimensionality reduction task, respectively

To evaluate the contribution of each task in UISNet for cancer outcome prediction, we compared the performance achieved by excluding different tasks from the framework. As shown in Fig. 4, when only the single prediction task was used to construct the neural network (-DRSS), the DNN framework achieved an average CI value of 0.610, which is 6.87% lower than that obtained by UISNet. When excluding the clustering task (-SS) from UISNet, the framework caused a decrease in the CI value from 0.655 to 0.631(− 3.66%), and a smaller decrease was caused by the exclusion of the dimensionality reduction task (-DR, CI = 0.642, − 1.98%). These results indicated that the clustering task provides more useful information than the dimensionality reduction task, and the prediction accuracy benefits from integrating these tasks into a unified framework.

### Independent tests

**Fig. 5:**
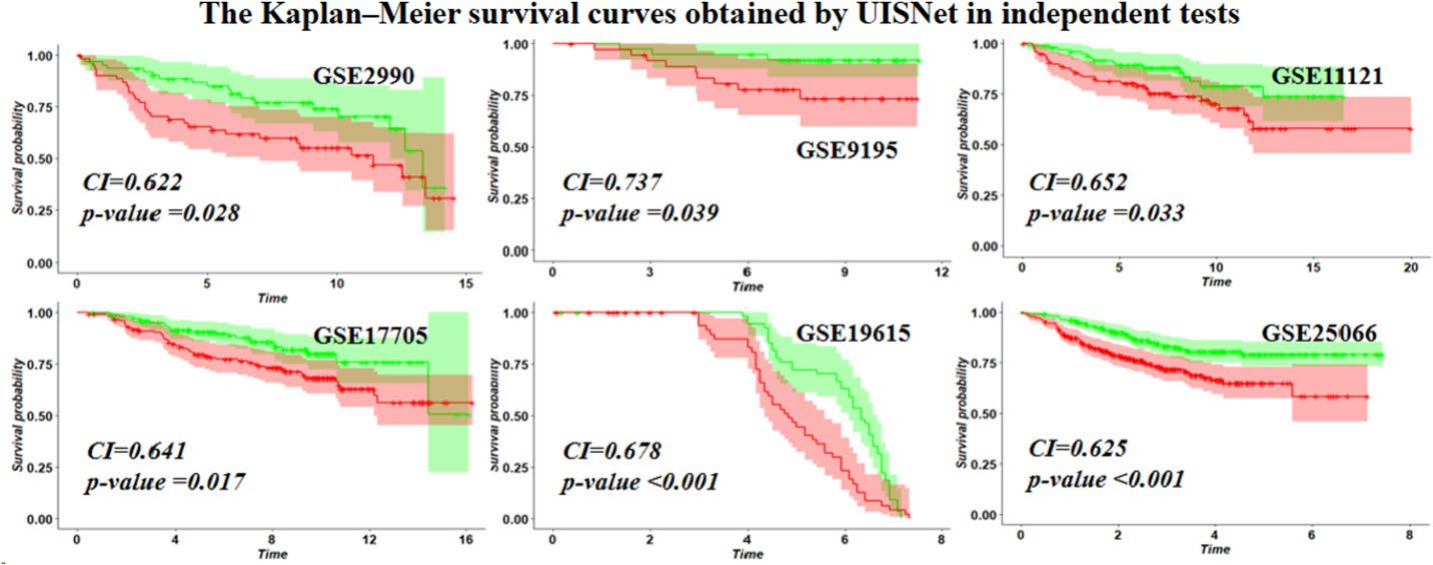
Kaplan‒Meier survival curves drawn based on the patient subgroups classified by UISNet. The red lines represent high-risk patients, and the black lines represent low-risk patients

To further validate the performance of the UISNet, we conducted independent tests by separating the breast cancer dataset from the integrated dataset BRCA_all as an independent test dataset (Fig. 5). The results indicated that the CI values obtained by UISNet in the independent tests are higher than 0.659, averagely. The Kaplan‒Meier survival curves illustrate the significant (P < 0.05) difference in survival between the two patient subgroups classified by UISNet. All these results proved the robustness of our method.

### Feature interpretability evaluation

**Fig. 6:**
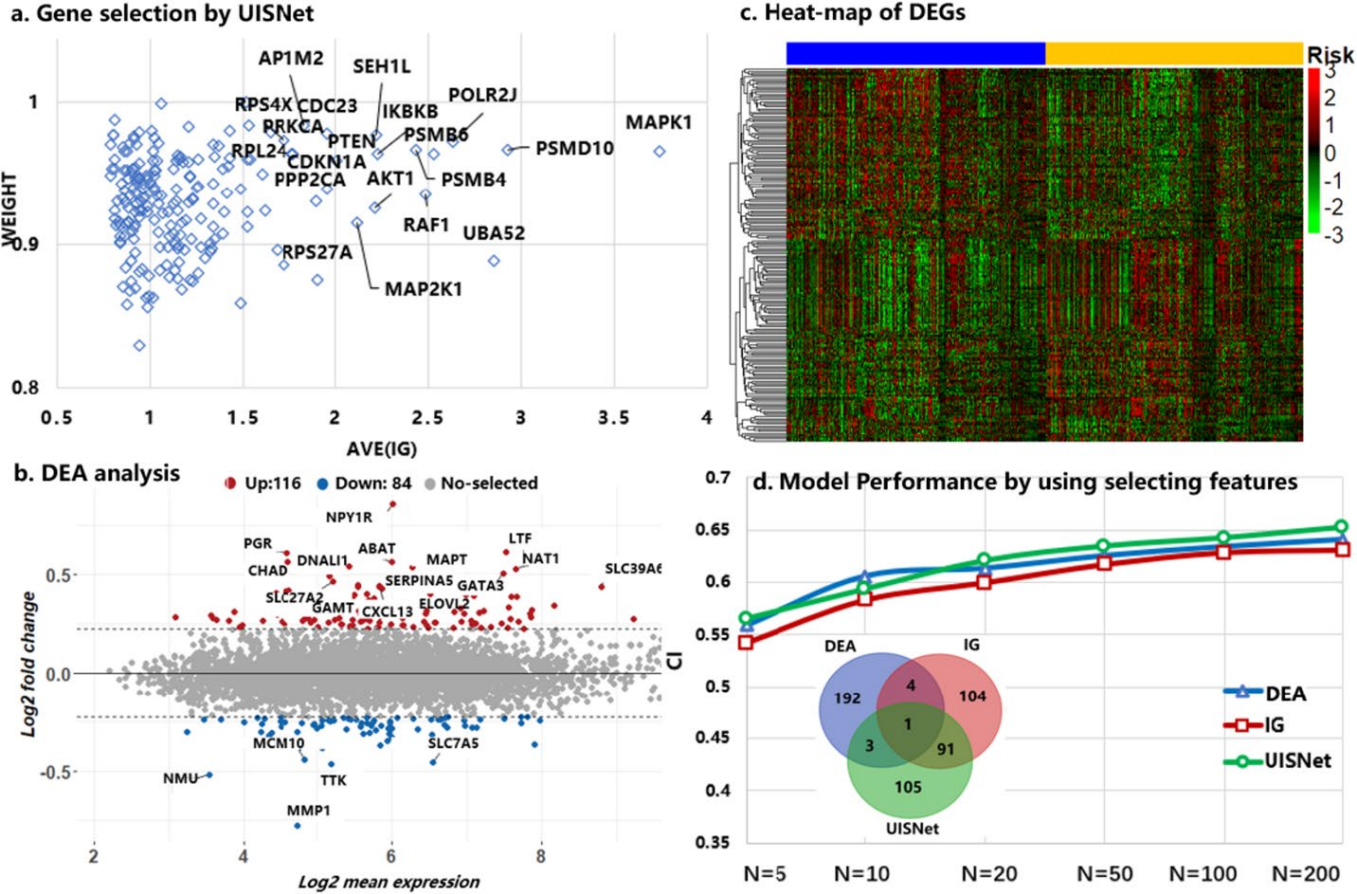
Breast cancer-related gene selection results produced by different methods. **a** Top 200 genes selected by computing the importance of the features in UISNet. The x-axis represents the average importance values of the different genes, and the y-axis is the uncertainty weight value computed by Eq. (12). **b** The results were used to identify the top 200 genes ranked based on the |log(fold change)| of DEA (adjusted-p-values < 0.05). **c** Heatmap of the 200 identified differentially expressed genes. **d** Breast cancer outcome prediction performance achieved by using different numbers of selected gene features based on DEA, the IG-based method without the uncertainty strategy (IG), and UISNet. A Venn diagram was used to show the number of overlapping genes selected by different methods

To evaluate the feature interpretability of UISNet, we compared UISNet with the method without using the uncertainty strategy (IG) and the differential expression analysis (DEA) while analyzing the BRCA_all dataset. The top 200 genes sorted based on the importance weights given by UISNet are shown in Fig. 6a. The IG values of these genes were calculated by Eq. (12). The result shows UISNet selected some important genes such as MAPK1, AKT1, RAF1, that have been proved related to breast cancer prognosis. Figure 6b shows the top 200 genes ranked based on the |log(fold change)| values produced by DEA (adjusted-p-values < 0.05), and the gene expression heatmap of these genes is shown in Fig. 6c. The names of the top 20 genes selected by UISNet and DEA are additionally annotated in Fig. 6a and b, respectively.

The Venn diagram in Fig. 6d shows the number of overlapping genes selected by different methods. The figure indicates that there are 92 overlapping genes selected from IG and UISNet, while the number of the common genes selected by DEA and UISNet is only four. Additionally, the breast cancer outcome prediction performance achieved by using different numbers of selected gene features based on DEA, IG, and UISNet are shown in Fig. 6d. It shows that while using the top 5 gene features, DEA and IG achieved lower CI values (0.559 and 0.542) than UISNet (0.565). When the number of used genes was 200, UISNet obtained the highest CI value of 0.652. The results demonstrate that by comparing with IG and DEA, UISNet can find genes that have a greater impact on the prognosis of breast cancer.

### Biointerpretability assessment

**Fig. 7:**
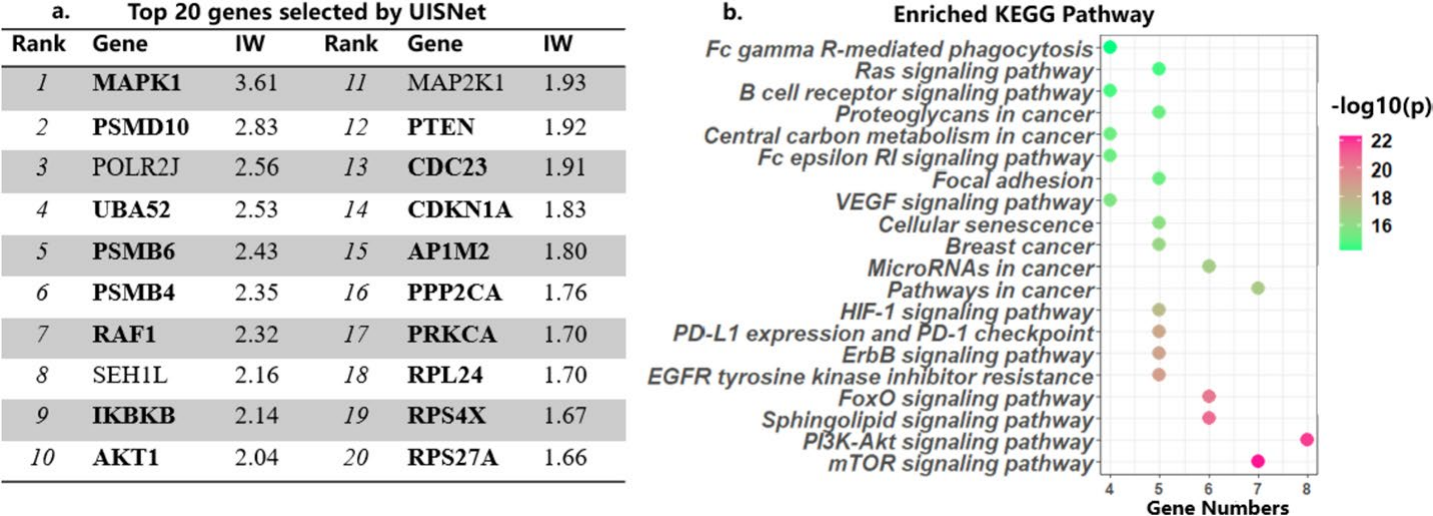
Interpretability assessment of the results obtained by UISNet. **a** The top 20 genes selected by UISNet ranked based on their importance weights. **b** The identified breast cancer-related KEGG pathways enriched by using the top 20 genes selected by UISNet

According to the importance weights (IWs) given by UISNet, we selected the top 20 genes for further analysis (Fig. 7a). Specifically, 17 of the selected genes have been validated to be associated with breast cancer. AKT1 encodes one of three human AKT serine-threonine protein kinase family members, and mutations in AKT1 are linked to breast cancer cell growth [37]. PTEN can negatively regulate intracellular phosphatidylinositol-3,4,5-triphosphate and exerts a tumor suppression effect by negatively regulating the PI3K-AKT signaling pathway [38]. In Lama’s study, the molecular changes in MAPK1 lead to overexpression of matrix metallopeptidase, which is associated with poor prognosis in breast cancer patients [39]. The upregulation of PRKCA has been found to be linked with resistance to antiestrogen treatment and the aggressive nature of tumors. PRKCA serves as a pivotal signaling hub and a potential therapeutic target in breast cancer stem cells, which exhibit comparable cell surface marker profiles to those observed in TNB [40]. CDC23, regulated by mir-34c, may be responsible for mir-34c-induced cell cycle arrest, where miR-34c can induce G2/M cell cycle arrest in breast cancer cells [41]. UBA52 has been reported to potentially associate with the development of resistance to Lapatinib in breast cancer treatment [42].

In addition, reduced expression of the RAF1 kinase inhibitor protein has been found to be associated with breast cancer metastasis [43]. The regulation of CDKN1A gene expression by LRH-1 influences the proliferation of breast tumor cells [44]. Downregulation of IKBKB expression by MicroRNA-16 enhances the sensitivity of breast cancer cells to paclitaxel treatment [45]. PPP2CA has been found to promote the proliferation and invasion of breast cancer cells [46]. Increased expression of PRKCA has been linked to resistance against antiestrogen treatment and the aggressive nature of tumors. Overexpression of PSMB4 promotes the proliferation and survival of breast cancer cells, lleading to an unfavorable prognosis [47]. The study revealed a significant upregulation in AP1M2, PSMD10, and RPL2 expression in breast cancer tissue compared to normal tissues [48–50]. RPS27A is overexpressed in breast cancer, enhanceing EBV-encoded LMP1-mediated proliferation and invasion through stabilization of LMP1 [51]. The RPS4X protein was identified as a potential biomarker for controlling cisplatin resistance in breast cancer treatment [52]. Although functional studies have not directly implicated MAP2K1, POLR2J, and SEH1L in breast cancer development and progression, they have been associated with other malignancies [53–55]. The indications from our model imply that they could potentially emerge as targets for breast cancer.

We further performed the KEGG enrichment analysis on these 20 genes (Fig. 7b), and found that they are enriched in many signaling pathways related to the occurrence and development of breast cancer, such as the mTOR and PI3K-AKT signaling pathways. The downstream transcription factors of the mTOR signaling pathway (with the highest enrichment score) include HIF1α, c-Myc, FoxO, and other important cancer regulatory molecules [56]. It has been proven that Paclitaxel can modulate the proliferation and migration of breast cancer cells via the mTOR signaling pathway [57]. The PI3K-AKT signaling pathway is a crucial component of many signaling pathways involving membrane-bound ligands, which are crucial for the survival of tumor cells [58]. The EGFR tyrosine kinase inhibitor resistance pathway enriched by UISNet shows that the identified genes may affect the tyrosine kinase inhibitor resistance in breast cancer treatment. The dysregulated activation of the ErbB signaling pathway plays a critical role in regulating cell growth, differentiation, and survival in breast cancer. Moreover, it is closely associated with tumor initiation, progression, and metastasis [59]. PD-L1 expression and PD-1 checkpoint pathway in breast cancer is closely related to immune regulation and tumor evasion from immune surveillance, which plays a key role in the regulation of immune responses [60]. The activation of the HIF-1 pathway in breast cancer is intricately associated with tumorigenesis, disease progression, and acquisition of treatment resistance [61]. Additionally identified genes enrich several breast cancer-related KEGG pathways, including VEGF and Ras signaling pathway, breast cancer pathway, pathways in cancer, and microRNAs in cancer. These findings demonstrate that UISNet can construct a breast cancer outcome prediction model with enhanced interpretability for biomedical applications.

## Discussion

Although our method provides model biological interpretability while improving the prediction accuracy, there are still some questions worth discussing, as described below. Firstly, the high censoring rates (12.17–87.03%) in the breast cancer data affected the calculation of the true survival proportion and decreased the performance of our method. Secondly, previous studies have shown that multi-omics integration is helpful for improving cancer outcome prediction performance. Expanding UISNet to integrate multi-omics data in an interpretable manner will be a potential way to improve the prediction performance of the model.

In the future, we will update our interpretable method by incorporating more medical information, including DNA methylation and slide images. Additionally, we want to design an effective strategy to evaluate the true survival times of censored patients to reduce the adverse impact of high censoring rates on model training. Furthermore, considering the molecular-level similarities observed between gynecologic and breast tumors [62], we will utilize the UISNet model to investigate gynecologic cancers such as cervical cancer and ovarian cancer, with the aiming of identifying potential pan-gynecologic-cancer related biomarkers for effective therapeutic interventions.

## Conclusions

DL-based methods have been proven to achieve accurate performance in cancer outcome prediction cases. Nevertheless, the lack of model interpretability limits the applicability of these methods. To address this challenge, we proposed a novel uncertainty-based interpretable deep neural network called UISNet for breast cancer outcome prediction. UISNet provides interpretable solutions by computing the integrated gradients of features with an uncertainty-based strategy. Furthermore, it improved model performance by introducing prior biological pathway knowledge and utilizing patient heterogeneity information. The experimental results show that UISNet achieved a 5.79% higher CI value than the compared state-of-the-art methods on average. Based on the feature interpretation results of the prediction model, 11 of the 20 identified genes have been proven to be associated with breast cancer. The comprehensive tests indicated that our proposed method is accurate and robust, and is an effective way to identify cancer-related genes. In summary, we believe that UISNet is a valuable and meaningful foundation for further cancer prognosis prediction research.

### Supplementary Information

**Additional file 1**. **Table S1**. The deviation obtained by UISNet in 5-fold cross validation and 10-fold cross validation.

## Data Availability

All the data analyzed are downloaded from GEO (https://www.ncbi.nlm.nih.gov) and TCGA (https://www.cancer.gov/about-nci/organization/ccg/research/structural-genomics/tcga). The method codes are available at https://github.com/chh171/UISNet.
