# Supplementary material for: An uncertainty-based interpretable deep learning framework for predicting breast cancer outcome
Source: BMC Bioinformatics. 2024 Feb 29;25:88. doi: 10.1186/s12859-024-05716-7 (PMC10902951; doi:10.1186/s12859-024-05716-7)
Supplement: Supplementary file 1 — Additional file 1. Table S1. The deviation obtained by UISNet in 5-fold cross validation and 10-fold cross validation. [file 12859_2024_5716_MOESM1_ESM.docx]

Table S1 gives the deviation obtained by UISNet in 5-fold cross validation and 10-fold cross validation in different datasets. The experiment shows that by computing the average C-index in different datasets, the values obtained in 5-fold cross validation (CI = 0.691) and 10-fold cross validation (CI = 0.695) by UISNet are close. The results provide evidence for the robustness of our method in different cross-validation.

Table S1. The deviation obtained by UISNet in 5-fold cross validation and 10-fold cross validation

| Dataset | 5-CV | 10-CV | Deviation |
| --- | --- | --- | --- |
| BRCA | 0.694  （$\pm$0.043） | 0.679  （$\pm$0.079） | 0.014 |
| GSE2990 | 0.596  （$\pm$0.043） | 0.602  （$\pm$0.114） | -0.006 |
| GSE9195 | 0.753  （$\pm$0.112） | 0.770  （$\pm$0.226） | -0.017 |
| GSE11121 | 0.727  （$\pm$0.074） | 0.724  （$\pm$0.131） | 0.003 |
| GSE17705 | 0.687  （$\pm$0.073） | 0.690  （$\pm$0.078） | -0.003 |
| GSE19615 | 0.703  （$\pm$0.048） | 0.718  （$\pm$0.076） | -0.015 |
| GSE25066 | 0.706  （$\pm$0.044） | 0.712  （$\pm$0.065） | -0.006 |
| BRCA_all | 0.660  （$\pm$0.031） | 0.665  （$\pm$0.039） | -0.005 |
| **Average** | 0.691 | 0.695 | -0.004 |
